# Efficient One-Pot Synthesis of 5-Chloromethylfurfural (CMF) from Carbohydrates in Mild Biphasic Systems

**DOI:** 10.3390/molecules18077675

**Published:** 2013-07-02

**Authors:** Wenhua Gao, Yiqun Li, Zhouyang Xiang, Kefu Chen, Rendang Yang, Dimitris S. Argyropoulos

**Affiliations:** 1 Departments of Chemistry and Forest Biomaterials, North Carolina State University, Raleigh, NC 27695-8005, USA; 2 State Key Laboratory Pulp and Paper Engineering, South China University of Technology, Guangzhou 510460, China; 3 Department of Chemistry, Jinan University, Guangzhou 510632, China; 4 Center of Excellence for Advanced Materials Research (CEAMR), King Abdulaziz University, P.O. Box 80203, Jeddah 21589, Saudi Arabia

**Keywords:** 5-chloromethylfurfural, carbohydrates, biphasic system

## Abstract

5-Halomethylfurfurals can be considered as platform chemicals of high reactivity making them useful for the preparation of a variety of important compounds. In this study, a one-pot route for the conversion of carbohydrates into 5-chloromethylfurfural (CMF) in a simple and efficient (HCl-H_3_PO_4_/CHCl_3_) biphasic system has been investigated. Monosaccharides such as D-fructose, D-glucose and sorbose, disaccharides such as sucrose and cellobiose and polysaccharides such as cellulose were successfully converted into CMF in satisfactory yields under mild conditions. Our data shows that when using D-fructose the optimum yield of CMF was about 47%. This understanding allowed us to extent our work to biomaterials, such as wood powder and wood pulps with yields of CMF obtained being comparable to those seen with some of the enumerated mono and disaccharides. Overall, the proposed (HCl-H_3_PO_4_/CHCl_3_) optimized biphasic system provides a simple, mild, and cost-effective means to prepare CMF from renewable resources.

## 1. Introduction

Locating new and versatile platform chemicals and biofuels from sustainable resources to replace those derived from petrochemicals is a central ongoing and urgent task prompted by depleting fossil fuel reserves and growing global warming concerns [1,2,3,4]. Alternative fine chemicals and biofuels that have been suggested to address some of these issues are butanol [5], ethanol [5], dimethylfuran [1], 5-ethoxymethylfurfural [2], γ-valerolactone, and alkanes produced from biomass [6,7]. Many of these alternatives rely on the efficient conversion of biomass carbohydrates into furfural derivatives. This is because biomass carbohydrates constitute 75% of the World’s renewable biomass and cellulose [4] and as such, they represent a promising alternative energy and sustainable chemical feedstock. In this regard, 5-halomethyfurfurals such as 5-chloromethylfurfural (CMF) and 5-bromomethylfurfural (BMF) has received significant attention as platform chemicals for synthesizing a broad range of chemicals and liquid transportation fuels [8,9].

CMF and BMF are extremely reactive [9] so that when subjected to further chemistries the provide a variety of important compounds for fine chemicals, pharmaceuticals, furan-based polymers and biofuels. These compounds include hydroxymethylfurfural (HMF) [9,10], 2,5-dimethylfuran (DMF) [1], and 5-ethoxymethylfurfural (EMF) [2], and some biologically active compounds [11]. Among them, DMF and EMF stand out since they possess excellent properties, including high energy density, high boiling point and water stability. For these reasons, they have been promoted as novel biofuels. In particular, EMF has been the subject of considerable attention since it possesses an energy density of 8.7 kWhL^−1^, substantially higher than that of ethanol (6.1 kWhL^−1^), and comparable to that of standard gasoline (8.8 kWhL^−1^) and diesel fuel (9.7 kWhL^−1^) [12]. Although, CMF and BMF themselves are not biofuels, they could readily be converted into EMF biofuels in ethanol in nearly quantitative yields.

The conventional synthesis of CMF involves the treatment of HMF or cellulose with dry hydrogen halide. More specifically, the hydroxyl group in HMF undergoes a facile halogen substitution reaction. Examples in the literature include those of Sanda *et al.* who obtained CMF from the reaction of ethereal gaseous hydrogen chloride with HMF [13]. Furthermore, while the conversion of cellulose into CMF was low (12%) [14,15], a substantially higher yield (48%) was obtained for the preparation of BMF when dry HBr was employed [16]. Considering the importance of these compounds, Mascal *et al.* recently reported the synthesis of CMF from cellulose treated by HCl-LiCl and successive continuous extraction [2]. Unfortunately, 5-(chloromethyl)furfural, 2-(2-hydroxyacetyl)furan, 5-(hydroxylmethyl), furfural and levulinic acid were also produced with this system. More recently, Kumari *et al.* reported the preparation of BMF from cellulose by a modified procedure using HBr-LiBr involving continuous extraction [17]. Despite the numerous efforts aimed at these transformations, each of them suffers from at least one of the following limitations: diverse by-products in significant yields that reduce the selectivity of the reaction and its economics, low conversions and yields, harsh reaction conditions (dry hydrogen halide, relative high temperature), requirements for large amounts of costly reagents (LiCl, LiBr), prolonged reaction times and tedious operations with complex set ups (continuous extraction) [18]. These drawbacks seriously hamper their potential industrial applications. Consequently, as part of our program aimed at developing new biofuels and fine chemicals based on biomass, we embarked our research for the development of efficient and economical methods aimed at converting carbohydrates to CMF under mild reaction conditions. 

In this communication we demonstrate the use of the biphasic mixture HCl-H_3_PO_4_/CHCl_3_ for the one-pot conversion of carbohydrates into CMF. The rational for the use of this biphasic approach is based on the thinking that as CMF is generated from HMF it is immediately transported and extracted from the aqueous acidic phase into the organic phase significantly minimizing by-product yields [9].

## 2. Results and Discussion

Initial efforts using the biphasic treatment (HCl(37%)-H_3_PO_4_(85%)/CHCl_3_) of the monosaccharide D-fructose at 45 °C for 20 h, offered a CMF yield of 35.8%. we then attempted to further optimize this result by systematically varying the volume ratio of HCl and H_3_PO_4_ (Table 1), the nature of the non-aqueous solvent, the reaction time and temperature. This is due to the fact that Table 1 deals only with the volume ratio of HCl and H_3_PO_4_. The effects of changing other variables are reported in Tables elsewhere.

**Table 1:** The effect various reaction variables on CMF yields from D-fructose ^a^.

**Entry**	**HCl/H_3_PO_4_ (v/v)**	**Temperature (°C)**	**Time (h)**	**Yields (mol%) ^b^**
1	1/0	45	20	28.4
2	2/1	45	20	36.9
3	3/1	45	20	42.1
4	4/1	45	20	46.8
5	5/1	45	20	45.5

^a^ D-fructose (5.0 mmol) was added in a mixture with specific volume ratio of 37% HCl and 85% H_3_PO_4_ (5.0 mL), and CHCl_3_ (5.0 mL). The system was stirred continuously at 45 °C for 20 h. ^b^ Isolated yields based on D-fructose.

The data of Table 1 indicates that the amount of hydrochloric acid in the biphasic medium plays an important role in this transformation. Increasing the amount of HCl in the system seemed to concomitantly increase the yield of CMF (Table 1, entries 1–5). Even at lower volume ratios of HCl/H_3_PO_4_ such as 2:1, the yield of CMF was seen to increase by 8.5%, compared with the reaction with just hydrochloric acid (Table 1, entries 1,2). H_3_PO_4_ offered enough hydrogen ions to promote the fructose selective conversion to CMF. The D-fructose molecule needs catalytic hydrogen ions to form fructofuranosyl intermediates, and the acid-induced elimination of three moles of water from this intermediate leads to the conversion to 5-hydroxymethyl-2-furaldehyde (HMF) [19,20]. As hydrochloric acid existed in the system, HMF could easily be transformed to 5-chloromethylfurfural (CMF). Increasing the mixed acid ratio (HCl/H_3_PO_4_) to 3:1 offered a CMF yield of 42.1% (Table 1, entry 3). Further increases in the mixed acid ratio (HCl/H_3_PO_4_) offered only marginal yield increases in the CMF yield (Table 1, entries 4,5). Therefore, in the dehydration reaction to produce CMF from fructose, HCl performed as a good mineral acid catalyst and provided the essential chloride for the production of CMF. Román-Leshkov *et al.* also observed various acid catalysts used to implement the dehydration reaction, while HCl showed the highest catalytic ability amongst common mineral acids [21].

The effect of reaction temperature was examined in detail by conducting CMF yield studies at 35, 45, and 55 °C at different ratios of HCl/ H_3_PO_4_ (Table 2).

**Table 2:** Effect of reaction temperature on the yields of CMF at different ratios of HCl/H_3_PO_4_^a^.

**Entry**	**HCl/H_3_PO_4_ (v/v)**	**Temperature (°C)**	**Time (h)**	**CMF Yield (mol %) ^b^**
1	3/1	35	20	38.5
2	3/1	45	20	42.1
3	3/1	55 ^c^	20	38.8
4	4/1	35	20	43.9
5	4/1	45	20	46.8
6	4/1	55	20	42.8
7	5/1	35	20	43.2
8	5/1	45	20	45.5
9	5/1	55	20	40.2

^a^ D-fructose (5.0 mmol) was added in a mixture with specific volume ratio of HCl(37%) and H_3_PO_4_(85%) (5.0 mL), and CHCl_3_ (5.0 mL). The system stirred continuously at the special temperature for 20h. ^b^ Isolated yields based on D-fructose. ^c^ Slightly below the CHCl_3_-water azeotrope boiling point (56.1 °C).

Upon increasing the temperature from 35 to 45 °C, the yields of CMF were seen to increase, when the mixed acid ratio of HCl/H_3_PO_4_ was varied from 3:1, 4:1 and 5:1 (Table 2, entries 1–9). At the temperature of 55 °C, however, all the CMF yields decreased somewhat compared to 45 °C (Table 2, entries 1–9). This could be rationalized on the basis of different energies of activation for the different side-reactions being more pronounced at the slightly higher temperatures.

In the presence of acid catalysts, fructose could dehydrate to produce CMF and various by-products. Increasing temperatures improved the yields of by-products. The cyclic fructofuransyl intermediate pathway degraded by means of secondary reactions, such as fragmentation, condensation or other dehydration reactions. In this respect our data was consistent with similar research by Moreau *et al.* [22] and Antal *et al.* [23] that dealt with the D-fructose dehydration mechanism and the formation of HMF. Overall, at lower reaction temperatures, the mixed acids did not offer their catalytic role, and the yield of CMF was not high. 

CMF yields obtained from the acid ratio (HCl/H_3_PO_4_) of 4:1 at different temperatures were seen to be higher than when the acid ratio was 3:1 or 5:1 (Table 2, entries 1–9). As such the acid volume ratio and the reaction temperature were established to be 4:1 and 45 °C, respectively, for this transformation.

The immiscible organic solvent plays an important role in extracting the products formed during the acidic aqueous phase reaction, thud significantly reducing side reactions. The nature of the extraction solvent was thus further explored and it was verified that indeed chloroform (Table 3, entry 1) was the extraction solvent of choice amongst all screened solvents. For example, 1, 2-dichlororethane (Table 3, entry 2) and 1-chlorobutane (Table 3, entry 3) were not able to extract all the CMF from the acidic aqueous phase, while toluene promoted a multitude of side reactions manifesting themselves in the complexity of the isolated product mixture. Unlike toluene, chloroform showed an excellent selectivity for extracting CMF from the acidic aqueous phase, simultaneously promoting reaction uniformity and reducing by-product diversity. Overall, this makes the work-up with chloroform and purification steps convenient with potential practical applications.

**Table 3:** Effect of extraction solvent on reaction yields ^a^.

**Entry**	**Extraction Solvent**	**Temperature (°C)**	**Time (h)**	**CMF Yield (mol%) ^b^**
1	Chloroform	45	20	46.8
2	1,2-Dichloroethane	45	20	28.8
3	1-Chlorobutane	45	20	22.6
4	Toluene	45	20	--- ^c^

^a^ D-fructose (5.0 mmol) was added in a mixture with 4/1 (v/v) of 37% HCl (4.0 mL) and 85% H_3_PO_4_ (1.0 mL), and organic solvent (5.0 mL ) and stirred continuously at 45 °C for 20 h. ^b^ Isolated yields based on D-fructose. ^c^ Complex mixture of compounds was formed as indicated by GC-MS.

Finally the effect of reaction time was investigated using the optimized reaction conditions determined so far. A 10 h reaction time offered a CMF yield of only 34.6% (Table 4, entry 1). Extending the reaction time to 20 h, dramatically increased the CMF yield by 12.2% (Table 4, entries 1,2). Further extension of the reaction time did not offer additional CMF yield improvements (Table 4, entries 3,4).

**Table 4:** Effect of reaction time on reaction yields ^a^.

**Entry**	**HCl/H_3_PO_4_ (v/v)**	**Extractant**	**Temperature (°C)**	**Time (h)**	**CMF Yield (mol%) ^b^**
1	4:1	CHCl_3_	45	10	34.6
2	4:1	CHCl_3_	45	20	46.8
3	4:1	CHCl_3_	45	30	44.9
4	4:1	CHCl_3_	45	40	45.2

^a^ D-fructose (5.0 mmol) was added in a mixture with 4/1 (v/v) of 37% HCl (4.0mL) and 85% H_3_PO_4_ (1.0 mL), and CHCl_3_ (5.0 mL ) and stirred continuously at 45 °C for special time. ^b^ Isolated yields based on D-fructose.

**Scheme 1:**
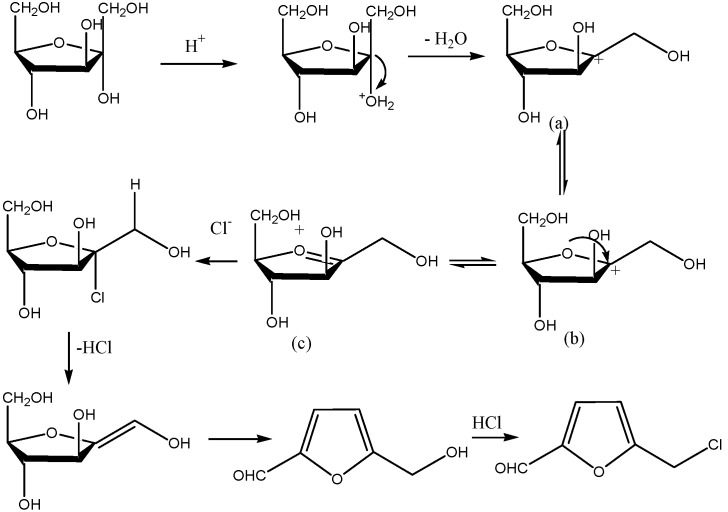
Possible route depicting the fructose dehydration to form CMF. Structures (a), (b) and (c) are fructofuransyl intermediates referred to in the text.

From the above analysis, a possible route of the fructose dehydration to form CMF as illustrated in Scheme 1 can be suggested.

In an effort to further understand and chart the selectivity and general applicability of the proposed methodology a series of mono-saccharine, two disaccharides and a poly-saccharide were examined. 

**Scheme 2:**
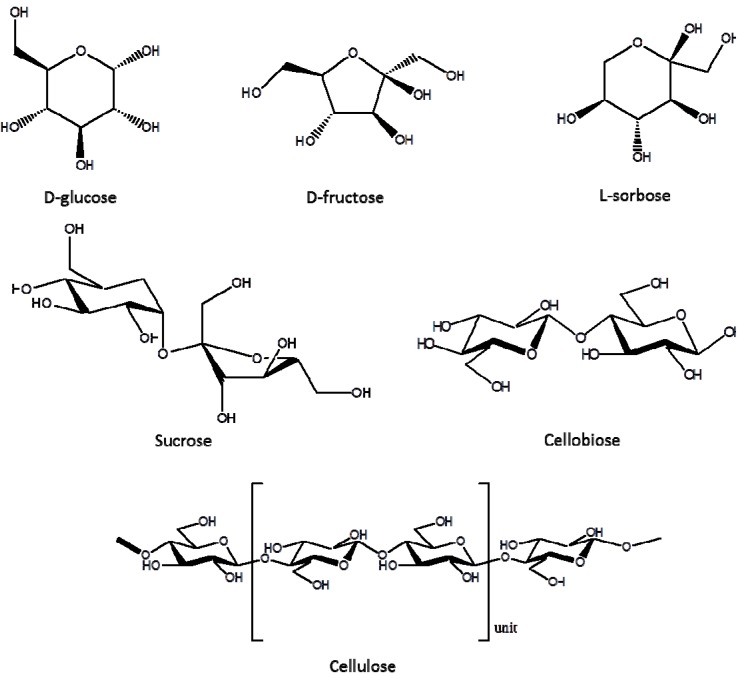
Structures of carbohydrates used and discussed in this work.

The data of Table 5 showed that fructose offered the highest yields of CMF (Table 5, entry 1), while glucose and cellulose the lowest (Table 5, entries 2–6). Interestingly D-fructose and D-glucose, while they are chemically very similar (Scheme 2), they produced significantly different yields of CMF (Table 5, entries 1,2). This is most likely because with the ketohexose (D-fructose) was easier to form the fructofuransyl intermediate than with the aldohexose (D-glucose), due to the higher reactivity of ketohexoses. It is likely that D-glucose could not dehydrate directly and transform into fructofuransyl intermediates under our conditions. The glucose to fructose isomerization reaction seems to be a pre-requisite for the formed fructose to dehydrate and yield CMF under the mixed acid catalytic system we propose. Our contention is supported by Rosatella *et al.* who proposed two methods to form the fructofuransyl intermediates, and the main one was the glucose to fructose isomerization reaction [24]. Furthermore, Zhao *et al.* [25] and Huang *et al.* [26] observed that in order to obtain high HMF yields from glucose the selective *in situ* isomerization of fructose was essential.

**Table 5:** Effect of different carbohydrate substrate on CMF yields ^a^.

**Entry**	**Carbohydrate**	**CMF Yield (mol %) ^b^**
1	Fructose	46.8
2	Glucose	7.3
3	Sorbose	16.4
4	Sucrose	43.1
5	Cellobiose	19.3
6	Cellulose	7.8

^a^ all reactions were performed in HCl-H_3_PO_4_/CHCl_3_ biphasic system at 45 °C for 20 h. ^b^ isolated yield based on carbohydrate.

The monosaccharide sorbose offered a better CMF yield than D-glucose (Table 5, entry 3), since it is a ketohexose. Although sorbose is one of the epimers (C-2 and C-3) of D-fructose (Scheme 2), it had similar reactivity to D-glucose. Our data is further supported by that of Khajavi *et al.* who has also documented that sorbose and glucose showed almost the same ability to produce HMF, but much lower than fructose [27]. 

The disaccharide sucrose (Scheme 2) comprised of linked fructose and glucose units afforded CMF yields similar to fructose (Table 5, entry 4, 43.1 mol%). In the mixed acid aqueous medium, the sucrose molecule was quickly hydrolyzed into fructose and glucose. Almost all of the fructose could be selectively converted into CMF in the biphasic system, due to the higher CMF yield from sucrose. Literature data also reports that 90% fructose solutions (obtained from sucrose hydrolysis) can be converted into fructofuransyl intermediates, while the glucose residue remains nearly unchanged [28]. Therefore, the overall activating nature of the carbonyl group in the C-2 position of the sugar seems to be apparent. Its absence (glucose and cellulose) dramatically reduces the reaction yields toward the production of CMF. 

Cellobiose consists of two glucose molecules joined by equatorial C1-C4 glycosidic bonds (Scheme 2). In an aqueous mixed acid solution, cellobiose was easily hydrolyzed to glucose molecules and as such it showed a somewhat higher CMF yield of 19.3% (Table 5, entry 5), while the polysaccharide cellulose only showed similar yields as glucose (Table 5, entry 6). The possible explanation for this observation was that cellulose is a high molecular weight linear polysaccharide, and its degradation involves the breaking of bonds between glucose units within the chain. As such the mixed acidic aqueous system might produce many random chain scissions, leading to many secondary reactions. Similar data have also been addressed in the literature by Emsley *et al.* [29] and Scheiding *et al.* [30]. 

Finally we examined various pulps and a wood powder for their potential to prepare CMF with the proposed methodology. Hardwood kraft pulp with the highest total sugar content (Table 6), containing mostly cellulose and hemicellulose, (which can hydrolyze into glucose and xylose) offered a yield of CMF of 21.3 mol% (based on the glucose of the pulp). This was a much higher than either glucose or cellulose could offer by themselves (Table 7, entry 1; Table 5, entries 2,6). The softwood (Norway spruce) thermomechanical pulp offered CMF yields of 22.8% while the hardwood powdered wood gave a CMF yield of 31.4% (both figures based on total sugar contents of these samples) (Table 7). These yields may be explained on the basis that mannose and other hexoses present in wood may also convert to fructofuransyl intermediates besides glucose. This data is supported by earlier efforts, where it was shown that mannose and glucose was of similar reactivity transforming to fructofuransyl intermediates producing hydroxymethylfurfural (HMF) [27]. Overall, our data with the complete lignocellulosic substrate is encouraging since all wood components were present during the reaction (cellulose, lignin and hemicelluloses). Under these circumstances, we believe that the obtained yields of CMF were encouraging. Efforts in our laboratory are continuing to further promote these yields in the presence of selective catalysts in the system [31].

**Table 6:** Sugar contents present in the examined lignocellulosic materials.

**Lignocellulose Sample**	**Rhamnose(%)**	**Arabinose(%)**	**Galactose(%)**	**Glucose(%)**	**Xylose(%)**	**Mannose(%)**	**Total Sugars (%)**
Eucalyptus Kraft pulp	0.0	0.0	0.0	75.9	20.8	0.0	85.7
Norway SpruceSoftwood TMP	0.0	0.0	1.8	44.2	6.3	11.8	64.1
Eucalyptus Hardwood	0.0	0.0	1.3	45.0	17.5	0.9	64.7

**Table 7:** Preparation of CMF with wood pulp and wood powder ^a^.

**Entry**	**Lignocellulose Sample**	**CMF Yield (mol %) ^b^**	**CMF Yield (mol %) ^c^**
1	Eucalyptus Kraft pulp	21.3	16.0
2	Norway Spruce softwood TMP	33.7	22.8
3	Eucalyptus hardwood	47.4	31.4

^a^ Lignocellulose sample (1.0 mg) was added in a mixture with 4/1 (v/v) of 37% HCl (4.0 mL) and 85% H_3_PO_4_ (1.0 mL), and CHCl_3_ (5.0 mL) and stirred continuously at 45 °C for special time at 45 °C for 20 h. ^b^ Isolated yield based on the glucose in the lignocellulose sample. ^c^ Isolated yield based on the total sugars in the lignocellulose sample.

## 3. Experimental

### 3.1. Materials and Instruments

All solvents and chemicals used were as obtained from commercial suppliers, unless otherwise indicated. ^1^H-NMR spectra were recorded on a Bruker Avance 300 instrument using CDCl_3_ as solvent and TMS as the internal standard. GC-MS spectra were performed on an HP G1800B GCD system.

*Eucalyptus globulus* wood powder and its ensuing kraft pulp were examined as well as a Norway Spruce sample of unbleached thermomechanical pulp, which was sampled in a Swedish mill of approximate 38% consistency and 85 mL Canadian Standard Freeness prepared by one-stage refining and a subsequent reject refining (about 20%) stage. All wood materials used in this work represent standard samples, being the subject of Cost action E 41 entitled; “Analytical tools with applications for wood and pulping chemistry” operated by the European Union. The sugar profiling for these materials was examined according to the procedure of Min *et al.* followed by ion chromatography (Dionex IC-3000; Dionex, Sunnyvale, CA, USA) (Table 6) [32,33].

### 3.2. General Procedure for the Synthesis of CMF from Model Carbohydrates

The selected carbohydrate (5.0 mmol) was added in a mixture of 37% HCl (4.0 mL), 85% H_3_PO_4_ (1.0 mL), and CHCl_3_ (5.0 mL) and it was stirred continuously at 45 °C for 20 h. Then an equal volume of water (5.0 mL) was added to quench the reaction. The reaction mixture was then extracted with CHCl_3_ 3 times. The combined organic extracts were then dried with anhydrous Na_2_SO_4_ for 4 h. Finally, the organic extracts were subjected to liquid chromatography (silica gel, CH_2_Cl_2_ as eluent) to offer the desired 5-chloromethylfurfural. The procedure of treating lignocellulose sample (Table 6) was almost the same as the carbohydrate, except adding the selected simple 1.0 mg each trial. The structure of 5-chloromethylfurfural was confirmed using ^1^H-NMR and GC-MS as follows: ^1^H-NMR (CDCl_3_): δ = 4.60 (s, 2H), 6.58 (d, *J* = 3.6 Hz, 1H), 7.18 (d, *J* = 3.6 Hz, 1H), 9.64 (s, 1H) ppm. GC-MS (EI, 80 eV): *m/z* 146 (M^+^, ^37^Cl, 10.53), 144 (M^+^, ^35^Cl, 32.0), 109 (C_6_H_5_O_2_^+^, 100), 81 (C_5_H_5_O^+^, 17.3).

## 4. Conclusions

In summary, this note describes an optimized biphasic system (HCl-H_3_PO_4_/CHCl_3_) that may pave the way for the development of a simple, mild, and cost-effective protocol for the conversion of various carbohydrates to CMF. The systematic optimization effort undertaken here delineates the structural features of carbohydrate residues that offer optimum CMF yields. Overall, the described procedure offers several advantages over other methodologies including mild reaction conditions; satisfactory product yields; and a simple experimental and isolation process. 

## Supplementary Materials

Supplementary File 1
