# Supplementary material for: Efficient One-Pot Synthesis of 5-Chloromethylfurfural (CMF) from Carbohydrates in Mild Biphasic Systems
Source: Molecules. 2013 Jul 2;18(7):7675–85. doi: 10.3390/molecules18077675 (PMC6269830; doi:10.3390/molecules18077675)
Supplement: Supplementary File 1 [file molecules-18-07675-s001.pdf]

Molecules **2014**, *19*, 1370-1374; doi:10.3390/molecules19011370

OPEN ACCESS

molecules

ISSN 1420-3049

www.mdpi.com/journal/molecules

Correction

Correction: Gao, W., *et al.* Efficient One-Pot Synthesis of 5-Chloromethyl-furfural (CMF) from Carbohydrates in Mild Biphasic Systems. *Molecules* 2013, *18*, 7675-7685

Wenhua Gao [1,2], Yiqun Li [3], Zhouyang Xiang [1], Kefu Chen [2], Rendang Yang [2] and Dimitris S. Argyropoulos [1,4,*]

1 Departments of Chemistry and Forest Biomaterials, North Carolina State University, Raleigh, NC 27695, USA

2 State Key Laboratory Pulp and Paper Engineering, South China University of Technology, Guangzhou 510460, China

3 Department of Chemistry, Jinan University, Guangzhou 510632, China

4 Center of Excellence for Advanced Materials Research (CEAMR), King Abdulaziz University, P.O. Box 80203, Jeddah 21589, Saudi Arabia

* Author to whom correspondence should be addressed; E-Mail: dsargyro@ncsu.edu; Tel.: +1-919-515-7708.

Received: 12 November 2013; in revised form: 13 January 2014 / Accepted: 13 January 2014 / Published: 22 January 2014

We have recently been made aware by Prof. Mark Mascal (University of California Davis) and the *Molecules* Editorial Offices of some errors and omissions in the Introduction section of our recent paper. The third paragraph of said Introduction currently reads as follows:

> *"The conventional synthesis of CMF involves the treatment of HMF or cellulose with dry hydrogen halide. More specifically, the hydroxyl group in HMF undergoes a facile halogen substitution reaction. Examples in the literature include those of Sanda et al. who obtained CMF from the reaction of ethereal gaseous hydrogen chloride with HMF [13]. Furthermore, while the conversion of cellulose into CMF was low (12%) [14,15], a substantially higher yield (48%) was obtained for the preparation of BMF when dry HBr was employed [16]. Considering the importance of these compounds, Mascal et al. recently reported the synthesis of CMF from cellulose treated by HCl-LiCl and successive continuous extraction [2]. Unfortunately, 5-(chloromethyl)furfural, 2-(2-hydroxyacetyl)-furan, 5-(hydroxymethyl)furfural and levulinic acid were also produced with this system. More recently, Kumari et al. reported the preparation of BMF from cellulose by a modified procedure using HBr-LiBr involving continuous extraction [17].*

Despite the numerous efforts aimed at these transformations, each of them suffers from at least one of the following limitations: diverse by-products in significant yields that reduce the selectivity of the reaction and its economics, low conversions and yields, harsh reaction conditions (dry hydrogen halide, relative high temperature), requirements for large amounts of costly reagents (LiCl, LiBr), prolonged reaction times and tedious operations with complex set ups (continuous extraction) [18]. These drawbacks seriously hamper their potential industrial applications. Consequently, as part of our program aimed at developing new biofuels and fine chemicals based on biomass, we embarked on research for the development of efficient and economical methods aimed at converting carbohydrates to CMF under mild reaction conditions".

To set straight the scientific record we would like to make the following corrections:

- Concerning the *sentences "Examples in the literature include those of Sanda et al. who obtained CMF from the reaction of ethereal gaseous hydrogen chloride with HMF [13]. Furthermore, while the conversion of cellulose into CMF was low (12%) [14,15], a substantially higher yield (48%) was obtained for the preparation of BMF when dry HBr was employed [16]."* We must now state that our references 14 and 15 were incorrect or irrelevant in this context. Furthermore, the yield mentioned for our reference 16 should be 56%, and not 48%. We attribute this last error to the fact that we used a figure (wrongly) quoted for this method in a secondary source, our reference 17, rather than the correct value given in the primary reference. Consequently, these sentences should be changed to: *"Examples in the literature include those of Sanda et al. who obtained CMF from the reaction of ethereal gaseous hydrogen chloride with HMF [13]. Additionally, Sanda et al. also developed a method of producing CMF by direct chlorination of HMF with Vilsmeier reagents [14]. Furthermore, while the conversion of cellulose into CMF was low (12%) [15], a substantially higher yield (56%) was obtained for the preparation of BMF when dry HBr was employed [16]."* with a new reference 15 as shown below:

  15. Fenton, H.J.H.; Gostling, M. LXXXV.—Derivatives of methylfurfural. *J. Chem. Soc. Trans.* **1901**, *79*, 807–816.

- While we cited a paper by Mascal *et al.* (our reference 2) and discussed some of the perceived shortcomings of that method, unfortunately in our literature search in preparation for writing this paper we missed a follow-up paper by the same authors that needs to be cited now as new reference 18 (see below), wherein the authors addressed all the problems we had identified for their method. This is an omission for which we apologize.

  18. Mascal, M.; Nikitin, E.B. Dramatic advancements in the saccharide to 5-(chloro-methyl)furfural conversion reaction. *ChemSusChem* **2009**, *2*, 859–861.

- We cited our original reference 18 in describing operations with complex set-ups, specifically mentioning continuous extraction as an example. While reference 18 does describe the preparation of CMF, that method involves a continuous flow reactor, and not continuous extraction, which was used in our references 2 and 17. Consequently reference 18 needs to be changed to new reference 18 and be cited along with references 2 and 17.

- In the reference section of the original text, references numbered 21 to 33 need to be renumbered as 19 to 31 and reference numbers 19 and 20 become references 32 and 33. This occurred due to a last minute rearrangement of our experimental section due to journal requirements that actually missed our critical reading of the galley proofs.

- Footnote (a) to Table 6 should state 1.0 g not 1.0 mg.

- The revised References section based on all these corrections is now provided below.

Revised References Section

1. Román-Leshkov, Y.; Barrett, C.J.; Liu, Z.; Dumesic, J.A. Production of dimethylfuran for liquid fuels from biomass-derived carbohydrates. *Nature* **2007**, *447*, 982–985.
2. Mascal, M.; Nikitin, E.B. Direct, High-yield conversion of cellulose into biofuel. *Angew. Chem. Int. Ed.* **2008**, *47*, 7924–7926.
3. Metzger, J.O. Production of liquid hydrocarbons from biomass. *Angew. Chem. Int. Ed.* **2006**, *45*, 685–698.
4. Corma, A.; Iborra, S.; Velty, A. Chemical routes for the transformation of biomass into chemicals. *Chem. Rev.* **2007**, *107*, 2411–2502.
5. Savage, D.F.; Way, J.; Silver, P.A. Defossiling fuel: How synthetic biology can transform biofuel production. *ACS Chem. Biol.* **2008**, *3*, 13–16.
6. Huber, G.W.; Chheda, J.N.; Barrett, C.J.; Dumesic J.A. Production of liquid alkanes by aqueous-phase processing of biomass-derived carbohydrates. *Science* **2005**, *308*, 446–450.
7. Bond, J.Q.; Alonso, D.M.; Wang, D.; West, R.M.; Dumesic, J.A. Integrated catalytic conversion of γ-valerolactone to liquid alkenes for transportation fuels. *Science* **2010**, *327*, 1110–1114.
8. Sanda, K.; Rigal, L.; Gaset, A. Optimisation of the synthesis of 5-chloromethyl-2-furancarboxaldehyde from D-fructose dehydration and *in-situ* chlorination of 5-hydroxymethyl-2-furancarboxaldehyde. *J. Chem. Technol. Biotechnol.* **1992**, *55*, 139–145.
9. Lewkowski, J. Synthesis, chemistry and applications of 5-hydroxymethylfurfural and its derivatives. *ARKIVOC* **2001**, *1*, 17–54.
10. James, O.O.; Maity, S.; Usman, L.A.; Ajanaku, K.O.; Ajani, O.O.; Siyanbola, T.O.; Sahu, S.; Chaubey, R. Towards the conversion of carbohydrate biomass feedstocks to biofuels via hydroxylmethylfurfural. *Energy Environ. Sci.* **2010**, *3*, 1833–1850.
11. Villain-Guillot, P.; Gualtieri, M.; Bastide, L.; Roquet, F.; Martinez, J.; Amblard, M.; Pugniere, M.; Leonetti, J.-P. Structure-activity relationships of phenyl-furanyl-rhodanines as inhibitors of RNA polymerase with antibacterial activity on biofilms. *J. Med. Chem.* **2007**, *50*, 4195–4204.

12. Gruter, G.J.M.; Dautzenberg, F. Method for the Synthesis of 5-Alkoxymethylfurfural Ethers and Their Use. EP Patent 1,834,950, A1, 2007.
13. Sanda, K.; Rigal, L.; Gaset, A. Synthesis of 5-bromomethyl-2-furancarboxaldehyde and 5-chloromethyl-2-furancarboxaldehyde. *Carbohydr. Res.* **1989**, *187*, 15–23.
14. Sanda, K.; Rigal, L.; Delmas, M.; Gaset, A. The Vilsmeier reaction: A new synthetic method for 5-(chloromethyl)-2-furaldehyde. *Synthesis* **1992**, *6*, 541–542.
15. Fenton, H.J.H.; Gostling, M. LXXXV.—Derivatives of methylfurfural. *J. Chem. Soc. Trans.* **1901**, *79*, 807–816.
16. Hibbert, H.; Hill, H.S. Studies on cellulose chemistry II. The action of dry hydrogen bromide on carbohydrates and polysaccharides. *J. Am. Chem. Soc.* **1923**, *45*, 176–182.
17. Kumari, N.; Olesen, J.K.; Pedersen, C.M.; Bols, M. Synthesis of 5-Bromomethylfurfural from cellulose as a potential intermediate for biofuel. *Eur. J. Org. Chem.* **2011**, *7*, 1266–1270.
18. Mascal, M.; Nikitin, E.B. Dramatic advancements in the saccharide to 5-(chloromethyl)furfural conversion reaction. *ChemSusChem* **2009**, *2*, 859–861.
19. Carlini, C.; Patrono, P.; Galletti, A.M.R.; Sbrana, G. Heterogeneous catalysts based on vanadyl phosphate for fructose dehydration to 5-hydroxymethyl-2-furaldehyde. *Appl. Catal. A-Gen.* **2004**, *275*, 111–118.
20. Benvenuti, F.; Carlini, C.; Patrono, P.; Galletti, A.M.R.; Sbrana, G.; Massucci, M.A.; Galli, P. Heterogeneous zirconium and titanium catalysts for the selective synthesis of 5-hydroxymethyl-2-furaldehyde from carbonhydrates. *Appl. Catal. A-Gen.* **2000**, *193*, 147–153.
21. Román-Leshkov, Y.; Chheda, J.N.; Dumesic, J.A. Phase modifiers promote efficient production of hydroxymethylfurfural from fructose. *Science* **2006**, *312*, 1933–1937.
22. Moreau, C.; Durand, R.; Razigade, S.; Duhamet, J.; Faugeras, P.; Rivalier, P.; Ros, P.; Avignon, G. Dehydration of fructose to 5-hydroxymethylfurfural over H-mordenites. *Appl. Catal. A-Gen.* **1996**, *145*, 211–224.
23. Antal, M.J., Jr.; Mok, W.S.L.; Richards, G.N. Mechanism of formation of 5-(hydroxymethyl)-2-furaldehyde from D-fructose and sucrose. *Carbohydr. Res.* **1990**, *199*, 91–109.
24. Rosatella, A.A.; Simeonov, S.P.; Frade, R.F.M.; Afonso, C.A.M. 5-Hydroxymethylfurfural (HMF) as a building block platform: Biological properties, synthesis and synthetic applications. *Green Chem.* **2011**, *13*, 754–793.
25. Zhao, H.; Holladay, J.E.; Brown, H.; Zhang, Z. Metal chlorides in ionic liquid solvents convert sugars to 5-hydroxymethylfurfural. *Science* **2007**, *316*, 1597–1600.
26. Huang, R.; Qi, W.; Su, R.; He, Z. Intergrating enzymatic and acid catalysis to convert glucose into 5-hydroxymethylfurfural. *Chem. Commun.* **2010**, *46*, 1115–1117.
27. Khajavi, S.H.; Kimura, Y.; Oomori, T.; Matsuno, R.; Adachi, S. Degradation kinetics of monosaccharides in subcritical water. *J. Food Eng.* **2005**, *68*, 309–313.
28. Carlini, C.; Giuttari, M.; Maria Raspolli Galletti, A.; Sbrana, G.; Armroli, T.; Busca, G. Selective saccharides dehydration to 5-hydroxymethyl-2-furaldehyde by heterogeneous niobium catalysts. *Appl. Catal. A-Gen.* **1999**, *183*, 295–302.
29. Emsley, A.M.; Stevens, G.C. Kinetics and mechanisms of the low-temperature degradation of cellulose. *Cellulose* **1994**, *1*, 26–56.

30. Scheiding, W.; Thoma, M.; Ross, A.; Schigerl, K. Modelling of the enzymatic hydrolysis of cellobiose and cellulose by a complex enzyme mixture of Trichoderma reesei QM 9414. *Appl. Microbiol. Biotechnol.* **1984**, *20*, 176–182.
31. Shi, N.; Liu, Q.; Wang, T.; Ma, L.; Zhang, Q. High yield production of 5-hydroxymethylfurfural from cellulose by high concentration of sulfates in biphasic system. *Green Chem.* **2013**, in press.
32. Min, D.; Li, Q.; Jameel, H.; Chiang, V.; Chang, H.M. Comparison of pretreatment protocols for cellulose-mediated saccharification of wood derived from transgenic low-xylan lines of cottonwood (*P. trichocarpa*). *Biomass Bioenergy* **2011**, *35*, 3514–3521.
33. Min, D.; Li, Q.; Jameel, H.; Chiang, V.; Chang, H.M. The cellulose-mediated saccharification on wood derived from transgenic low-lignin lines of black cottonwood (*Populus trichocarpa*). *Appl. Biochem. Biotechnol.* **2012**, *168*, 947–955.

- Finally, in summarizing we would like to offer the following rewrite of the Conclusions section of our paper:

In summary, this note describes an optimized biphasic system (HCl-H3PO4/CHCl3) that may pave the way for the development of a mild, and cost-effective protocol for the conversion of various carbohydrates to CMF. The systematic optimization effort undertaken here delineates the structural features of carbohydrate residues that may eventually offer optimum CMF yields.

The authors would like to apologize to the readership of *Molecules* for these errors, and welcome this opportunity to correct the scientific record.

© 2014 by the authors; licensee MDPI, Basel, Switzerland. This article is an open access article distributed under the terms and conditions of the Creative Commons Attribution license (http://creativecommons.org/licenses/by/3.0/).
